# The epidemiological characteristics and effectiveness of countermeasures to contain coronavirus disease 2019 in Ningbo City, Zhejiang Province, China

**DOI:** 10.1038/s41598-021-88473-4

**Published:** 2021-05-05

**Authors:** Xuying Lao, Li Luo, Zhao Lei, Ting Fang, Yi Chen, Yuhui Liu, Keqin Ding, Dongliang Zhang, Rong Wang, Zeyu Zhao, Jia Rui, Yuanzhao Zhu, Jingwen Xu, Yao Wang, Meng Yang, Bo Yi, Tianmu Chen

**Affiliations:** 1 State Key Laboratory of Molecular Vaccinology and Molecular Diagnostics, School of Public Health, Xiamen University, 4221-117 South Xiang’an Road, Xiang’an District, Xiamen, Fujian Province People’s Republic of China; 2 Ningbo Municipal Center for Disease Control and Prevention, 237 Yongfeng Road, Haishu District, Ningbo City, Zhejiang Province People’s Republic of China

**Keywords:** Infectious diseases, Applied mathematics

## Abstract

A novel coronavirus (SARS-CoV-2) has spread worldwide and led to high disease burden around the world. This study aimed to explore the key parameters of SARS-CoV-2 infection and to assess the effectiveness of interventions to control the coronavirus disease 2019 (COVID-19). A susceptible—exposed—infectious—asymptomatic—recovered (SEIAR) model was developed for the assessment. The information of each confirmed case and asymptomatic infection was collected from Ningbo Center for Disease Control and Prevention (CDC) to calculate the key parameters of the model in Ningbo City, China. A total of 157 confirmed COVID-19 cases (including 51 imported cases and 106 secondary cases) and 30 asymptomatic infections were reported in Ningbo City. The proportion of asymptomatic infections had an increasing trend. The proportion of elder people in the asymptomatic infections was lower than younger people, and the difference was statistically significant (Fisher’s Exact Test, *P* = 0.034). There were 22 clusters associated with 167 SARS-CoV-2 infections, among which 29 cases were asymptomatic infections, accounting for 17.37%. We found that the secondary attack rate (SAR) of asymptomatic infections was almost the same as that of symptomatic cases, and no statistical significance was observed (*χ*^2^ = 0.052, *P* = 0.819) by Kruskal–Wallis test. The effective reproduction number (*R*_*eff*_) was 1.43, which revealed that the transmissibility of SARS-CoV-2 was moderate. If the interventions had not been strengthened, the duration of the outbreak would have lasted about 16 months with a simulated attack rate of 44.15%. The total attack rate (TAR) and duration of the outbreak would increase along with the increasing delay of intervention. SARS-CoV-2 had moderate transmissibility in Ningbo City, China. The proportion of asymptomatic infections had an increase trend. Asymptomatic infections had the same transmissibility as symptomatic infections. The integrated interventions were implemented at different stages during the outbreak, which turned out to be exceedingly effective in China.

## Introduction

The coronavirus disease 2019 (COVID-19), with the pathogen of severe acute respiratory syndrome coronavirus 2 (SARS-CoV-2), has spread worldwide and led to significant disease burden around the world, especially in China, South Korea, Japan, Iran, and the United States of America^1–5^. As of 14 February 2021, there have been 108,153,741 confirmed cases globally and 101,515 in China, reported to WHO^6^. Rapid increase in the number of confirmed cases, wide range of countries affected, and enormous impact on people’s health and national economies prioritized the importance of understanding the epidemiological characteristics and the transmission mechanism of COVID-19.

As the most affected country in the first wave of epidemic transmission, it is essential to assess the effectiveness of interventions implemented in China during the outbreak and to share out the experience of disease control for other countries to prepare for the possible following wave(s) of the outbreak. As a commonly employed tool to assess the effectiveness of interventions, mathematical models have been used to explore the epidemiological characteristics and the transmission mechanism of COVID-19, forecasting of the pandemic transmission and assess the effectiveness of interventions such as social distance and wearing masks^1,7–10^. However, certain key parameters of COVID-19 remain unclear, such as the proportion and the transmissibility of the asymptomatic infections, which might lead to some uncertainty of the modelling results.

A susceptible—exposed—infectious—asymptomatic—recovered (SEIAR) model was developed based on our previous studies^7,11,12^. In this study, we employed the SEIAR model to fit the data of SARS-CoV-2 symptomatic and asymptomatic infections in the city, and aimed to calculate the key parameters (including the proportion and the transmissibility of the asymptomatic infections), and further to assess the effectiveness of interventions implemented in Ningbo City, Zhejiang Province, China.

## Methods

### Ethics statement

This study was designed and performed according to the Helsinki declaration and was approved by the Ethical Review Committee of Ningbo Municipal Center for Disease Control and Prevention (No. 202001). Informed consents were obtained from all participants, while parenteral or legal guardian was also included in. For participants who were less than 18 years of age, consents were obtained from their parents or legal guardians. All data was analyzed anonymously.

### Data collection

Ningbo City is one of the large cities in Zhejiang Province, China, with a population of 8.2 million. In this study, the information of all the reported COVID-19 confirmed cases and asymptomatic infections in Ningbo City as of February 25, 2020, including sex, age, occupation, exposed date, onset date, and diagnosed date was collected from Ningbo Center for Disease Control and Prevention (CDC). Contact tracing of each case was performed to investigate the number of close contacts, as well as the contact modes and time. The case investigation was conducted according to the criteria of the National Novel Coronavirus Pneumonia Prevention and Control Program announced by National Health Commission of the People’s Republic of China.

### Case definitions and case finding

COVID-19 was classified as suspected case, confirmed case, and asymptomatic infection as follows:

- Suspected case: A suspected case could be diagnosed if there is an epidemiological history plus any 2 of the clinical features, or no clear epidemiological history but with 3 of the following clinical features: fever and/or respiratory symptoms; showing specific imaging features of COVID-19. In the early stage of the disease, the total number of leukocytes was normal or decreased, and the lymphocyte count was decreased.
- Confirmed case: It should be a suspected case with any one of the following evidences: SARS-CoV-2 was detected by real-time fluorescence reverse transcription-polymerase chain reaction (RT-PCR); the genome of the virus was sequenced and highly homologous to the known new coronavirus.
- Asymptomatic infection: No symptom but SARS-CoV-2 was tested positive from respiratory tract specimens.

Close contacts are defined as: During the period from two days before the symptoms onset to the day when a confirmed case is isolated, people who have close contact (within 1 m) with the confirmed case without effective protection, such as those people who live, study, work together with the patients; medical staff, family members or other people who have close contact during the diagnosis, treatment, nursing of and visiting to the patients; passengers who share the same vehicle and have close contact during the trip with the cases. Contact tracing of each case was performed by epidemiological investigators.

### Specimen collection and the virus testing

Sample collection: the respiratory tract specimens (such as throat swab, nasal swab, deep expectoration fluid, respiratory tract aspirate, bronchial lavage fluid, alveolar lavage fluid, etc.) were collected at the early stage of infection. The specimens were then repacked in the biosafety cabinet of the secondary biosafety laboratory. All specimens were placed in a suitable size sample collection tube with a spiral cover and a gasket. The information of the collected samples was recorded, including the sample number, sample type, patient’s name, and sampling date, before the specimens were sent to the laboratory within 24 h for detection. The collection, transportation and detection of specimens were conducted according to the second category of highly pathogenic microorganisms to ensure the biological safety.

The nucleic acid of the COVID-19 virus was detected by real-time fluorescence RT-PCR. Two pairs of novel coronavirus gene primers and probes were selected for ORFlab and N gene (ORFlab: Forward primer 5′-CCCTGTGGGTTTTACACTTAA-3′, Reverse primer 5′-ACGATTGTGCATCAGCTGA-3′, Fluorescence probe 5′-FAM-CCGTCTGCGGTATGTGGAAAGGTTATGG—BHQl-3′; N: Forward primer 5′-GGGGAACTTCTCCTGCTAGAAT-3′, Reverse primer 5′-CAGACATTTTGCTCTCAAGCTG-3′, Fluorescence probe 5′-FAM-TTGCTGCTGCTTGACAGATT-TAMRA-3′). Nucleic acid was extracted by using virus RNA/DNA nucleic acid extraction reagent of Tianlong biology. Real-time fluorescent RT-PCR was conducted by using novel coronavirus SAR-CoV-2 nucleic acid reagents of Shanghai Berger. The reaction system was referred to the instructions of relevant manufacturers. Result judgement: negative, means no CT value or CT value > 40; positive means CT value < 37; suspicious means CT value is between 37 and 40, and it is recommended to repeat the experiment.

### Transmission model

Based on our previous study^7,13^, we have constructed a multi-population and multi-path dynamic model of crowd propagation, considering the actual situation in Ningbo City, this study adopts the "human-to-human" (person-to-person, PP) transmission model. The PP model refers to SEIAR model. In the model, individuals were divided into five compartments: susceptible (*S*), exposed (*E*), infectious (*I*), asymptomatic (*A*) and recovered (*R*). The model is based on following assumptions and facts:

- During outbreak period, natural birth rate and death rate of population were at a relative low level and therefore not considered in the model.
- Importation of COVID-19 cases was due to people’s mobility. We had collected the data of imported cases as well, the importation was simulated by a function as follows:
  $$Importation={n}_{t}$$
  In this function, *n*_*t*_ refers to imported COVID-19 cases at time *t*.
- The incubation period and latent period of human infection was defined as 1/*ω* and 1/*ω’*. Based on our previous study^7^, we set *ω* = *ω’*.
- The infectious period of *I* and *A* was defined as 1/*γ* and 1/*γ’*. By analyzing the reported data, we found that *I* and *A* were both isolated when they were diagnosed in Ningbo City. Therefore, we set *γ* = *γ’*.
- No death case was reported in Ningbo City. Therefore, case fatality rate was no considered in our study.
- The proportion of asymptomatic infections was defined as *p*.
- The *S* would be infected through sufficient contact with *I*, and the transmission rate was defined as *β*. We also assumed that the transmissibility of *A* was *κ* times that of *I*, where 0 ≤ *κ* ≤ 1.

Therefore, the SEIAR model is shown as follows:

$$\left\{\begin{array}{l} \\ \frac{dS}{dt}=-\beta S\left(I+A\right) \\ \frac{dE}{dt}=\beta S\left(I+kA\right)-\omega E \\ \frac{dI}{dt}=\left(1-p\right)\omega E-\gamma I \\ \frac{dA}{dt}=p\omega E-\gamma A \\ \frac{dR}{dt}=\gamma I+\gamma A\end{array}\right.$$

### The transmissibility of the COVID-19 based on the SEIAR model

Commonly, *R*_0_ was defined as the expected number of secondary infections that result from introducing a single infected individual into an otherwise susceptible population^14–16^. If *R*_0_ > 1, the outbreak will occur. If *R*_0_ < 1, the outbreak will go toward an end. Therefore, *R*_0_ = 1 is the threshold of transmission. However, *R*_0_ is used to describe the transmissibility of disease under ideal conditions. If intervention was implemented, *R*_0_ should be replaced as effective reproduction number (*R*_*eff*_) which could be calculated by the following equation^17^:

$${R}_{eff}=\frac{\beta S\left(1-p+\kappa p\right)}{\gamma }$$

### Parameter estimation

Parameters of the SEIAR model were shown in Table 1. In our previous study, we found that the epidemic curve was be heterogeneous before and after the implementation of interventions^15,16^. Therefore, the values of *β* should be different in different stages during the transmission. In our study, we estimated the values of *β* by curve fitting of the SEIAR model with the reported data.

**Table 1:** Description and values of parameters in SEIAR model.

Parameter	Description	Unit	Value	Range	Methods
*β*_1_	Transmission rate at stage 1	Individuals^−1^·days^−1^	5.81 × 10^–8^	> 0	Curve fitting
*β*_2_	Transmission rate at stage 2	Individuals^−1^·days^−1^	8.87 × 10^–10^	> 0	Curve fitting
*κ*	Relative transmissibility rate of *A* to *I*	1	1	0–1	Reported data and references^22^
*p*	Proportion of *A*	1	0.1737	0–1	Reported data and references^20,21^
*ω*	Incubation relative rate	days^−1^	0.2	0.0667–1	Reported data and references^19^
*ω'*	Latent relative rate	days^−1^	0.2	0.0667–1	Reported data and references^19^
*γ*	Removed rate of the infectious	days^−1^	0.3333	0.0667–1	Reported data
*γ'*	Removed rate of the asymptomatic infections	days^−1^	0.3333	0.0667–1	Reported data

We calculated the secondary attack rate (SAR) of *A* and *I* from clustered cases in our collected data. The existing research results and the difference between SAR of *A* and *I* were then combined and tested to define the value of *κ*.

In this study, we calculated the value of *p* from clustered cases in Ningbo City. We also collected the date of exposure, date of symptom onset, and date of case isolation. Therefore, the values of *ω*, *ω’*, *γ*, and *γ’* were estimated by data collected and combined with existing research results.

### Simulation methods and statistical analysis

The imported cases were simulated as transmission sources and the secondary cases were employed for the curve fitting. Berkeley Madonna 8.3.18 (developed by Robert Macey and George Oster of the University of California at Berkeley. Copyright ©1993–2001 Robert I. Macey & George F. Oster, CA, USA) was employed to perform the procedures of curve fitting and the simulation. The simulation methods (Runge–Kutta method of order four with tolerance set at 0.001) were the same as the previously published researches^11,12,14–16,18^. The goodness of fit was judged by Chi-square (χ^2^) value calculated by SPSS 21.0 (IBM Corp, Armonk, NY, USA). Epidemiological characteristics analysis was also performed by SPSS 21.0 (IBM Corp, Armonk, NY, USA). Differences in epidemiological characteristics of COVID-19 were analyzed by two side way tests.

## Results

### Epidemiological characteristics

**Figure 1:**
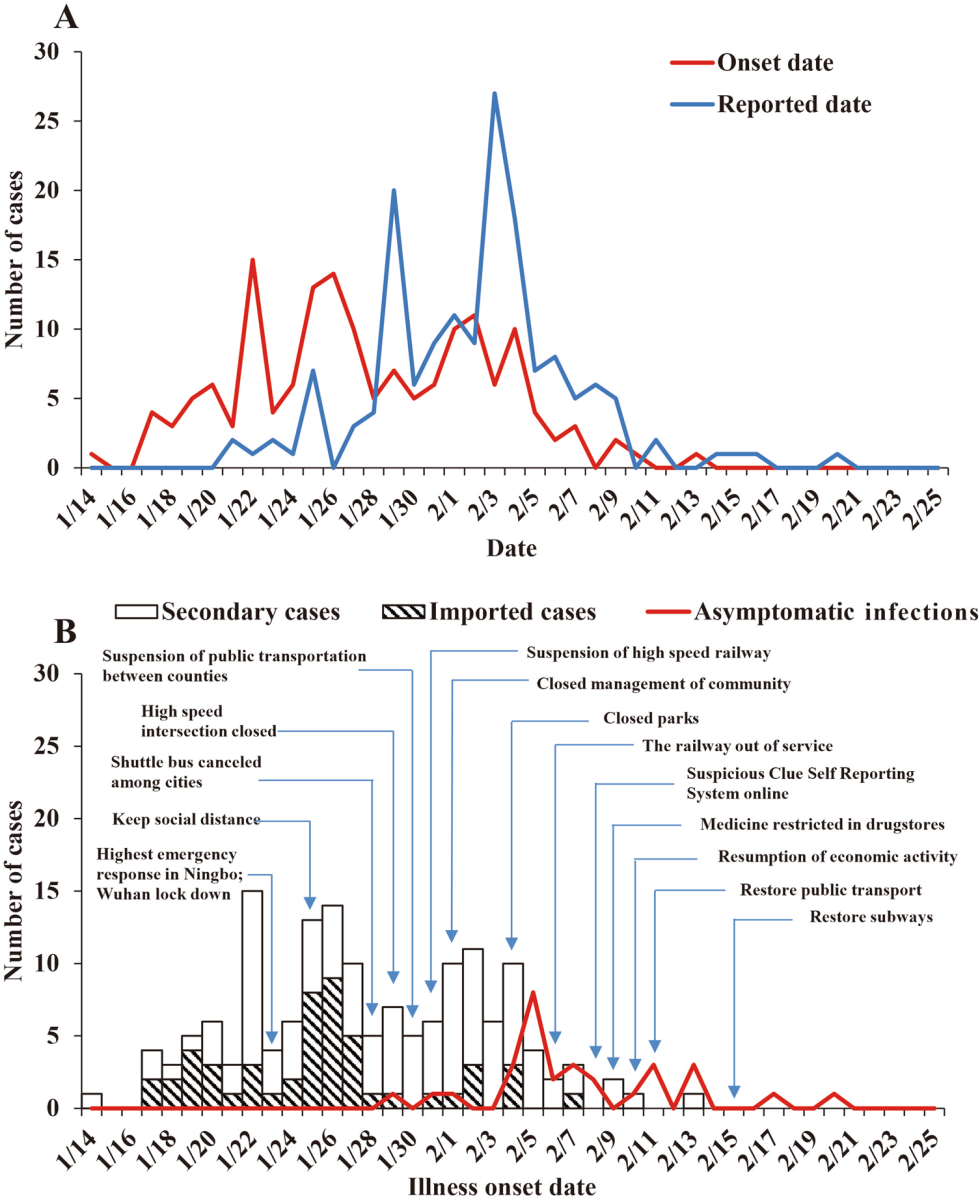
Epidemic curve of reported cases and interventions of COVID-19 in Ningbo City, Zhejiang Province, China. (A) Epidemic curve of reported cases; (B) Imported cases, secondary cases and asymptomatic infections among reported cases, and interventions of COVID-19.

**Figure 2:**
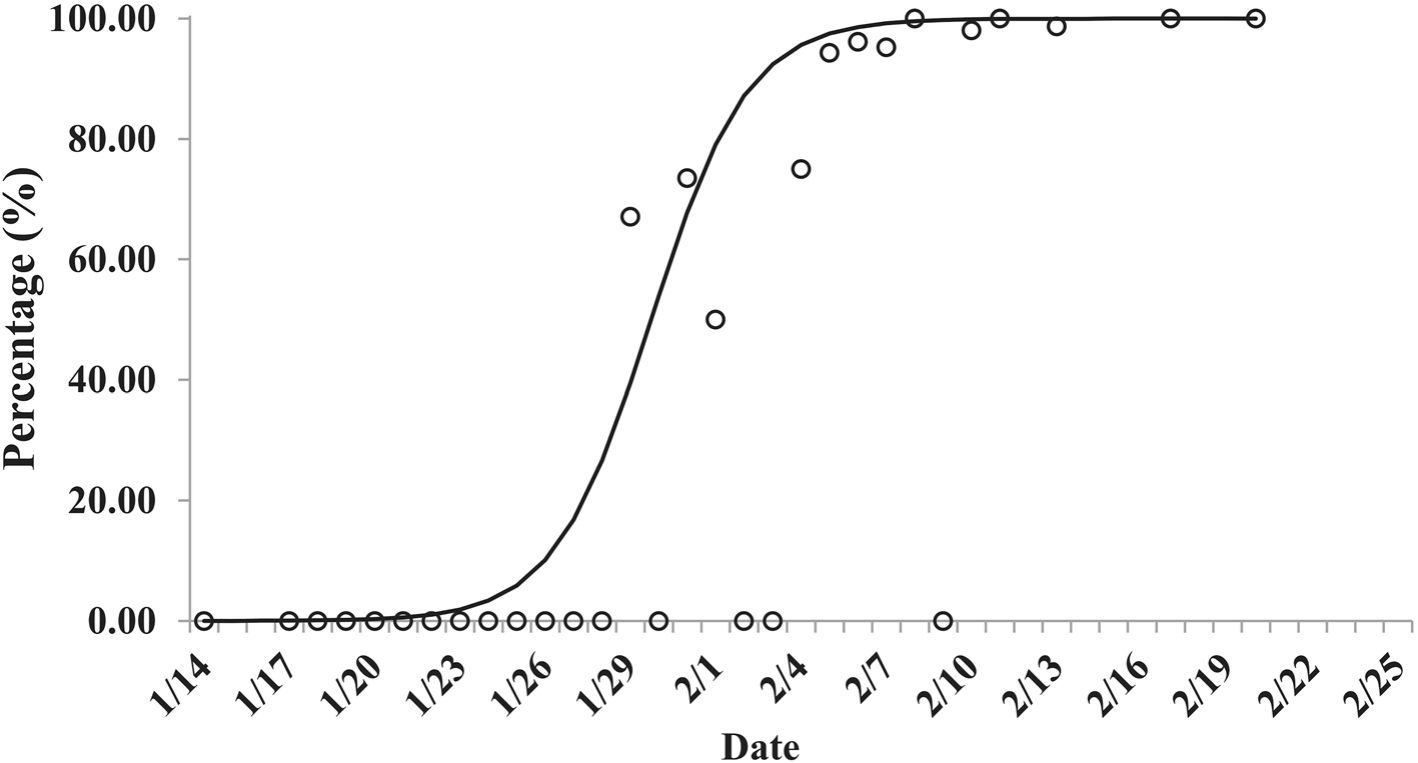
The temporal distribution of the proportion of asymptomatic infections of COVID-19 in Ningbo City, Zhejiang Province, China.

As of February 25, 2020, a total of 157 confirmed COVID-19 cases (including 51 imported cases and 106 secondary cases) and 30 asymptomatic infections were reported in Ningbo City. The peak of the reported cases was on January 22(onset date), and February 3, 2020(reported date), respectively (Fig. 1-A). The peak of the imported cases, secondary cases, and asymptomatic infections occurred on January 26, January 22, and February 5, 2020, respectively (Fig. 1-B). The proportion of asymptomatic infections had an increasing trend and fitted well with a logistic differential equation model (Fig. 2).

The incidence of female was higher than that male, while the proportion of asymptomatic infections of female was lower than male, but no statistical significance was observed (*χ*^2^ = 2.196, *P* = 0.138). The incidence of elder people was higher than that younger people, while the proportion of asymptomatic infections of elder people was lower than younger people, and the difference was statistically significant (Fisher’s Exact Test, *P* = 0.034). The most infected people were farmers, housework and unemployment individuals, retirees, public officials, and students. However, the scatter children, workers, students, individual business people, housework and unemployment individuals, and farmers had the highest proportion of asymptomatic infections(Table 2).

**Table 2:** Epidemiological characteristics of reported COVID-19 cases in Ningbo City, Zhejiang Province, China.

Characteristics	Number of symptomatic cases	Number of asymptomatic infections	*p *(%)	*IR* (per 100 000)
**Sex**				
Male	56	15	21.13	1.71
Female	101	15	12.93	2.86
**Age (years)**				
0–9	1	3	75.00	0.59
10–19	4	2	33.33	1.00
20–29	13	3	18.75	1.44
30–39	27	4	12.90	2.16
40–49	20	5	20.00	1.69
50–59	47	9	16.07	4.24
60–69	29	1	3.33	3.14
70–79	11	3	21.43	3.52
≥ 80	5	0	0.00	2.30
**Occupation**				
Scattered children	1	1	50.00	NA
Workers	8	4	33.33	NA
Students	10	4	28.57	NA
Individual business people	3	1	25.00	NA
Houseworker and unemployed	36	7	16.28	NA
Farmers	36	7	16.28	NA
Business services	17	3	15.00	NA
Retirees	22	3	12.00	NA
Public officials	11	0	0.00	NA
Teachers	6	0	0.00	NA
Migrant workers	1	0	0.00	NA
Security staffs	1	0	0.00	NA
Lawyers	1	0	0.00	NA
Business owners	1	0	0.00	NA
Medical staffs	1	0	0.00	NA
Unknown	2	0	0.00	NA

*p* proportion of asymptomatic cases, *IR* infection rate, *NA* not available.

**Figure 3:**
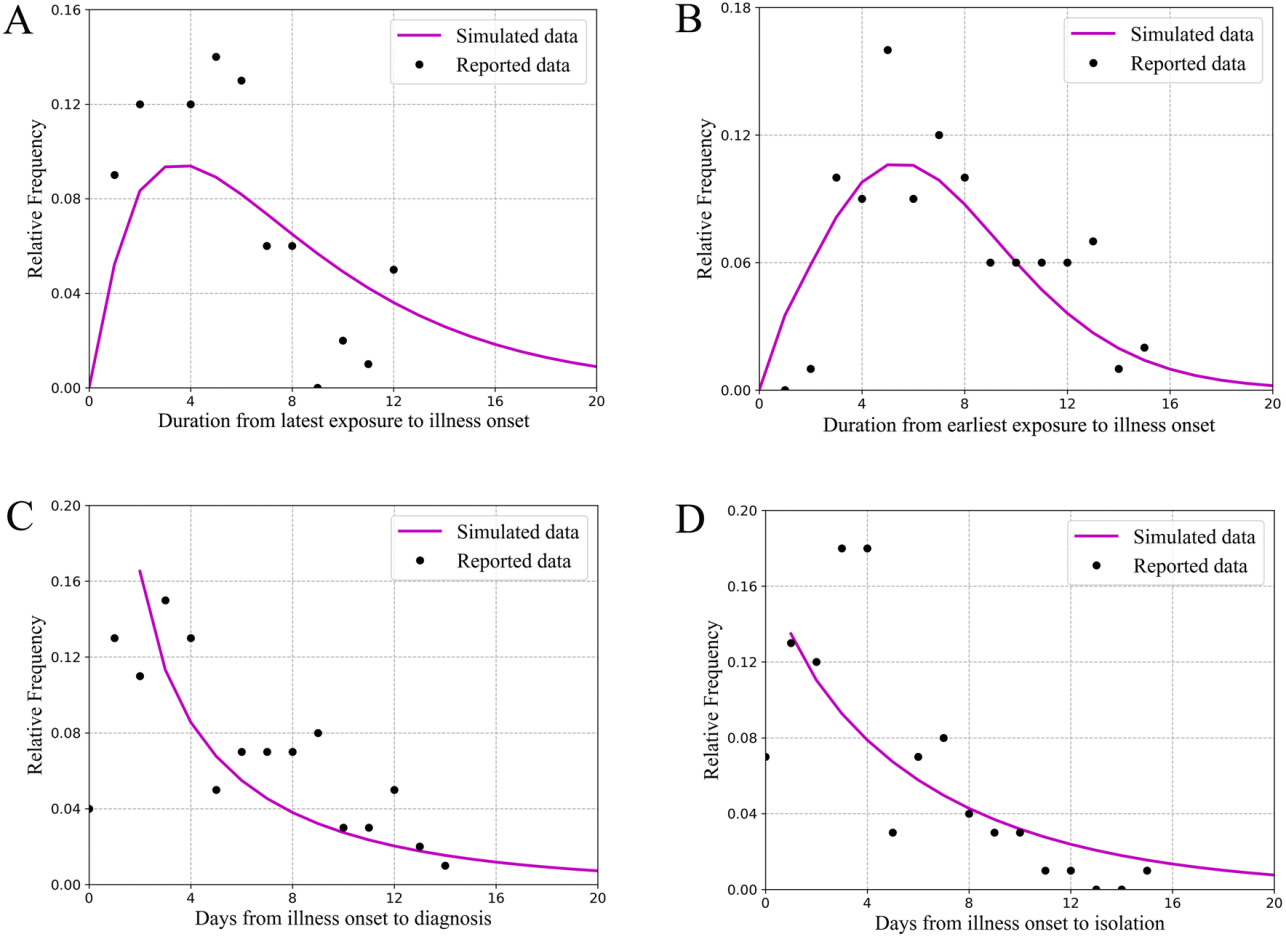
Key time-to-event distributions. The estimated short incubation period distribution (i.e., the time from latest exposure to illness onset) is shown in Panel (**A**). The estimated long incubation period distribution (i.e., the time from earliest exposure to illness onset) is shown in Panel (**B**). The estimated distribution of times from illness onset to diagnosed date is shown in Panel (**C**). The estimated distribution of times from illness onset to isolated date is shown in Panel (**D**).

The median incubation period of the reported cases was 5 days (range 1–15 days) (Fig. 3). Therefore, *ω* = *ω’* = 0.2, this value is also close to some other research results^1,7,19^. The median duration from symptoms onset to diagnosed date and from symptoms onset to isolation of the reported cases was 4.0 days (range 0–17 days) and 3.0 days (range 0–15 days), respectively (Fig. 3). Therefore, *γ* = *γ’* = 0.3333.

The range of the proportion of asymptomatic infections was from 0.1518 to 0.2340^20,21^, and as of February 25, 2020, there were 22 clusters associated with 167 SARS-CoV-2 infections reported in Ningbo City, among which 167 clustered infections, 29 cases were asymptomatic infections, with a proportion of 17.37%,thus *p* = 0.1737. The infection sources of 2 clusters were found to be asymptomatic (denoted as Cluster A) and the other 20 clusters were symptomatic (denoted as Cluster B). We found that the SAR of asymptomatic infections was almost the same as that of symptomatic cases, and no statistical significance was observed (*χ*^2^ = 0.052, *P* = 0.819) by Kruskal–Wallis test. There is also a research claimed that no statistical significant difference between the transmissibility of asymptomatic infections versus that of symptomatic cases^22^. Therefore, *κ* = 1. We also found that the proportion of asymptomatic infections in the infected cases was higher in Cluster A than Cluster B (Table 3).

**Table 3:** The secondary attack rates in twenty-two clusters of COVID-19 in Ningbo City, Zhejiang Province, China.

Outbreak ID	Classification of infection source	Basic information of the infection source		Number of close contacts	Number of secondary cases		SAR (%)
		Sex	Age (years)		Symptomatic	Asymptomatic	
A	Asymptomatic	Male	24	48	2	1	6.25
B	Asymptomatic	Male	12	41	1	1	4.88
C	Symptomatic	Female	64	1347	66	15	6.01
D	Symptomatic	Male	54	128	3	1	3.13
E	Symptomatic	Male	31	44	1	0	2.27
F	Symptomatic	Male	37	11	2	0	18.18
G	Symptomatic	Female	49	6	1	0	16.67
H	Symptomatic	Male	49	87	1	2	3.45
I	Symptomatic	Female	54	86	4	0	4.65
J	Symptomatic	Female	58	3	1	1	66.67
K	Symptomatic	Male	45	27	4	0	14.81
L	Symptomatic	Male	29	17	3	0	17.65
M	Symptomatic	Male	62	104	5	0	4.81
N	Symptomatic	Female	66	101	7	0	6.93
O	Symptomatic	Male	50	38	6	1	18.42
P	Symptomatic	Male	56	88	1	1	2.27
Q	Symptomatic	Female	71	8	1	0	12.50
R	Symptomatic	Female	35	22	1	0	4.55
S	Symptomatic	Female	38	36	0	2	5.56
T	Symptomatic	Female	63	4	2	0	50.00
U	Symptomatic	Female	29	41	5	2	17.07
V	Symptomatic	Female	57	191	1	0	0.52

*SAR* secondary attack rate.

### Transmissibility of COVID-19

**Figure 4:**
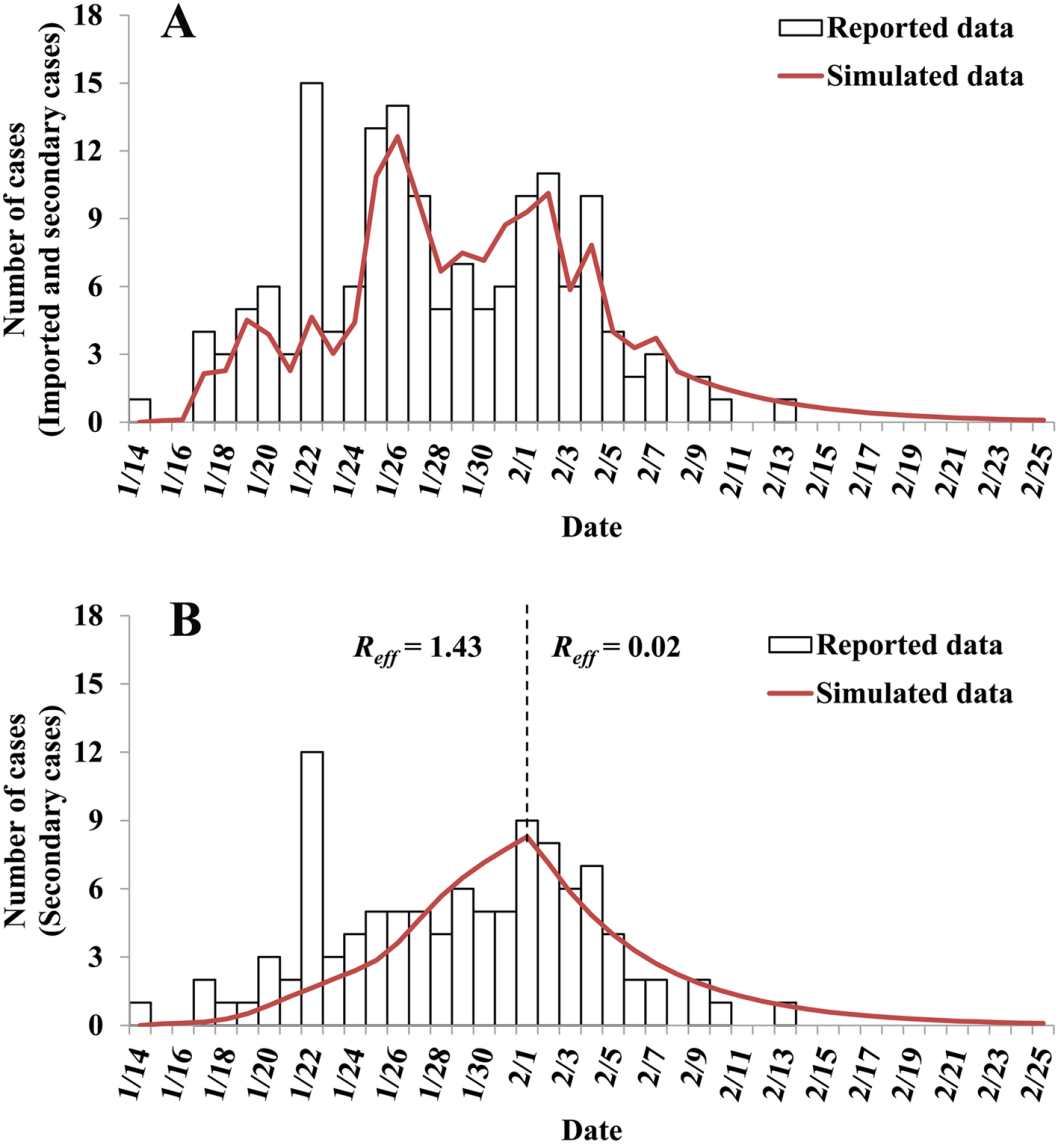
Results of curve fitting of the SEIAR model with the reported data. (**A**) curve fitting with imported and secondary cases; (**B**) curve fitting with secondary cases.

The simulated results showed that our model fitted well (*χ*^2^ = 39.798, *P* = 0.524) with the reported epidemic curve of COVID-19 in Ningbo City (Fig. 4). The data were divided into stage 1 (before February 1, 2020) and stage 2 (after February 1, 2020), and the parameter *β* was divided into *β*_1_ and *β*_2_, whose values of them were 5.81 × 10^–8^ and 8.87 × 10^–10^, respectively. Therefore, the values of *R*_*eff*_ were 1.43 and 0.02 in these two stages.

### Effectiveness of countermeasures

**Figure 5:**
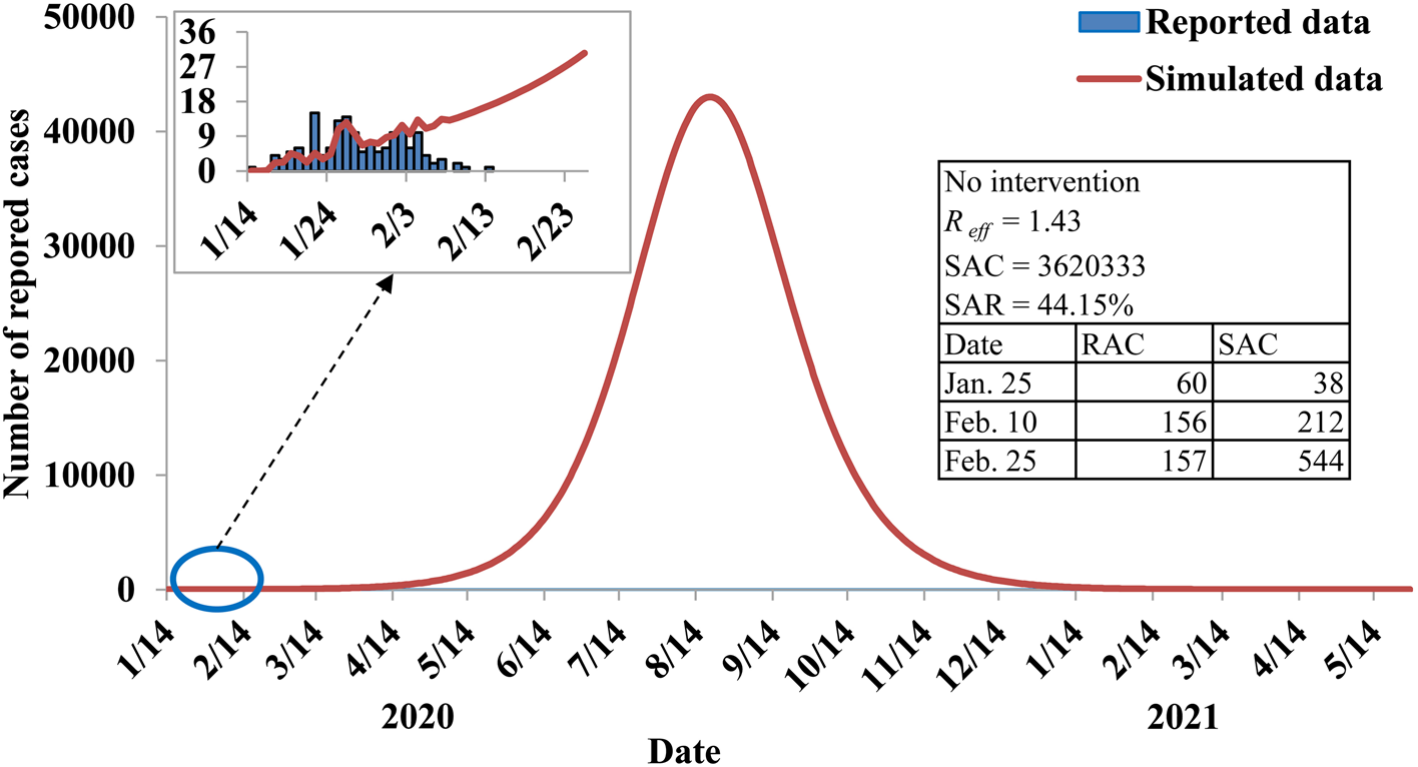
Effectiveness of countermeasures implemented in Ningbo City, Zhejiang Province, China. *R*_*eff*_ effective reproduction number, *SAC* simulated accumulative cases, *SAR* simulated attack rate, *RAC* reported accumulative cases.

Based on our simulation, if interventions had not been strengthened on February 1, 2020, the SARS-CoV-2 would have spread rapidly with a peak on August 19, 2020 and the reported COVID-19 cases would reach 43,011. Moreover, the transmission would have lasted on May 25, 2021. The duration of the outbreak would have lasted about 16 months with a simulated attack rate of 44.15% (Fig. 5). In Ningbo City, the integrated interventions were implemented at different stages during the outbreak (Fig. 1), which turned out to be exceedingly effective (Table 4).

**Table 4:** Effectiveness of interventions implemented at different time.

	*R*_*eff*_ = 1.43			*R*_*eff*_ = 2.20			*R*_*eff*_ = 3.58		
	Number of cases	TAR (%)	DO (days)	Number of cases	TAR (%)	DO (days)	Number of cases	TAR (%)	DO (days)
Reported data	157	0.0019	31	157	0.0019	31	157	0.0019	31
*t* = 30	343	0.0042	49	1302	0.0159	59	13,499	0.1646	73
*t* = 60	1812	0.0221	88	51,285	0.6254	109	4,375,366	53.3581	130
*t* = 90	8233	0.1004	126	1,492,328	18.1991	155	6,553,084	79.9157	141
*t* = 120	36,086	0.4401	163	5,268,580	64.2510	182	6,564,749	80.0579	147
*t* = 150	153,075	1.8668	201	5,695,610	69.4587	197	6,564,851	80.0592	153
*t* = 180	584,939	7.1334	237	5,716,242	69.7103	212	6,564,852	80.0592	154
*t* = 210	1,642,857	20.0348	270	5,717,231	69.7223	227	6,564,852	80.0592	154
*t* = 240	2,811,023	34.2808	299	5,717,278	69.7229	242	6,564,852	80.0592	154
*t* = 270	3,379,328	41.2113	324	5,717,281	69.7229	242	6,564,852	80.0592	154
*t* = 300	3,556,361	43.3703	347	5,717,281	69.7229	242	6,564,852	80.0592	154
No intervention	3,620,333	44.1504	497	5,717,281	69.7229	242	6,564,852	80.0592	154

If January 14, 2020 was set as the initial time (*t* = 0), we simulated the interventions were strengthened on different length of delay (*t* = 0, 30, 60, …) (Table 4). We found that total attack rate (TAR) and duration of the outbreak (DO) would increase when the length of delay increased. But these two indices increased rapidly when the length of delay was longer than 180 days. Similar findings were also observed when we increased the values of *R*_*eff*_ up to 2.20 and 3.58 (Table 4). TAR would increase up to 69.72% and 80.06%, and DO would decline down to 242 days and 154 days, respectively. These two indices increased rapidly when the length of delay was longer than 90 days and 60 days.

## Discussion

In our study, we analyzed the SAR of asymptomatic infections and symptomatic cases, and found that the value of *κ* was 1, which suggest the transmission potential of asymptomatic infections is the same as those of symptomatic cases. The result is consistent with the findings of Guangdong Provincial CDC^23^. This finding is fire-new in Zhejiang Province and indicates that it is necessary to take effective isolation measures for asymptomatic infections seriously and cautiously, such measures can greatly reduce further spread of the virus. If isolation measures are not compulsorily applied to asymptomatic patients, they would not have the awareness to seek health care or visit hospital and thus could not be identified in their infectious period, the virus would then be spread to other close contacts^24,25^.

Amid the global outbreak of COVID-19, many scientists speculate that some infected people could be highly contagious even when they are mild or asymptomatic, and a growing number of studies have shown that many COVID-19 patients with no or merely mild symptoms could transmit the virus to other people. About 17.9% of the 634 infected people on the Diamond Princess cruise ship never showed any symptoms^26^. A research team in Japan reported that 13 of the 565 Japanese citizens evacuated from Wuhan in early February, 2020 were infected, with four of them were asymptomatic infections^27^. The viral load detected in asymptomatic patients was similar to that in symptomatic patients^23^. A new study shows that at least 50% of SARS-CoV-2 infection cases come from asymptomatic people^28^. In our study, among the 167 clustered infections, 29 cases were asymptomatic infections, taking up 17.37%. Due to the differences among regions, the proportion of asymptomatic patients was likely to be higher than we thought. We also found that the proportion of asymptomatic cases showed an increasing trend. The reason of such increase remains unclear, which in our perspective might be: (a) although asymptomatic infections were difficult to detect at the early phase of outbreak, when the specialists explored the virus more deeply and thoroughly, the capacity to detect and diagnose asymptomatic infections would be improved; (b) with the concern of virus evolution, the pathogenicity is likely to be weakened with generations during transmission, therefore there may be an rising trend of asymptomatic infections.

,*R*_*eff*_ 1.43 and 0.02 respectively. Compared with our previous study ^7^ it is noticeable that the transmissibility of SARS-CoV-2 in Ningbo City is at a moderate level, which of course, *R*_*eff*_ rather than basic *R*_0_,^29^,^30^.

Similar findings were observed when we simulated the values of *R*_*eff*_ up to 2.20 and 3.58, the results showed that TAR and DO would raise along with the increasing length of delay of interventions or with no inventions. The higher the *R*_*eff*_ value, the higher the TAR value is. However, regardless of the transmission capacity is, a series of timely prevention and control measures, such as closed community management, maintaining social distance and so on, executed by the government, could reduce the effective reproduction number and finally be helpful to contain the outbreak. It suggested that effective and timely interventions played an important role in controlling the outbreak, such result was similar to other researches^31,32^.

Based on the fitting results of the model, we determined that the intervention measures taken by the government of Ningbo City are remarkably effective, while without other evaluation process by ground truth data, such as case–control study to verify the authenticity of the results. The reason is that during the COVID-19 period, such tests are not ethical. This actually reflects the substitutability of mathematical modeling, and many related studies use mathematical models to explore the effectiveness of interventions^33–35^. In response to the currently discovered SARS-CoV-2 variants infections, although the data analyzed in this study was from the early stage of the outbreak in Ningbo, the model is also applicable to variants infections, just the parameters are different. The corresponding parameters can be modified in the future for further research.

In this study, the median incubation period of the reported cases was 5.0 days, the median duration from symptoms onset to diagnosed date and from symptoms onset to isolation of the reported cases was 4.0 days and 3.0 day, respectively. The results are similar to another recent estimate^36^. Another research showed the median incubation period was 6.7 days, the interval time from illness onset to being diagnosed was 4.5 days^37^. The difference in period might be related to the different diagnostic capacity and virus transmission range in each region.

Ningbo City was successful in preventing and controlling the COVID-19 infection. It is largely benefited from timely and effective interventions. Firstly, Wuhan City was locked down on January 23, 2020, which decreased the imported cases and therefore reduced the infection sources in Ningbo City. Secondly, Ningbo City implemented measures such as social distancing, suspension of the subways, highways, and airlines, staying at home, and wearing masks in a timely manner, which strongly cut off most of the transmission routes. In addition, the city was strict in enhancing the surveillance system, case finding, and testing the suspected cases or even close contacts, which made sure that symptomatic and asymptomatic infections were isolated timely, thus greatly reducing the spread of virus.

## Limitations

Our research still has certain limitations. The effectiveness of interventions was assessed as a combination of comprehensive interventions, rather than as single intervention. However, one of the research purposes of this study is to evaluate the effectiveness of intervention measures in Ningbo City, instead of evaluating the effectiveness of a single prevention and control measure. Therefore, the results of this study can still achieve our research goals. More data are urgently needed to accurately estimate the effectiveness of single interventions in the future.

## Conclusions

SARS-CoV-2 displayed moderate transmissibility in Ningbo City, Zhejiang Province, China. The proportion of asymptomatic infections had an increase trend. Asymptomatic infection has the same transmissibility as symptomatic one. The integrated interventions were implemented at different stages during the outbreak, which turned out to be extremely effective to control COVID-19 spread in Ningbo City. If interventions had not been strengthened in time, the transmission would have lasted for 16 months and more than 44% of people would have been infected.

## Data Availability

Extra data is available by emailing to Bo Yi (yibonb@163.com) on reasonable request.

## Abbreviations

COVID-19: Coronavirus disease 2019
SARS-CoV-2: Severe acute respiratory syndrome coronavirus 2
SEIAR: Susceptible—exposed—infectious—asymptomatic—recovered
CDC: Center for disease control and prevention
RT-PCR: Reverse transcription-polymerase chain reaction
SAR: Secondary attack rate
TAR: Total attack rate
DO: Duration of the outbreak
